# Method for Rapid Protein Identification in a Large Database

**DOI:** 10.1155/2013/414069

**Published:** 2013-08-13

**Authors:** Wenli Zhang, Xiaofang Zhao

**Affiliations:** ^1^Institute of Computing Technology, Chinese Academy of Sciences, Beijing 100190, China; ^2^State Key Laboratory of Computer Architecture, ICT, CAS, Beijing 100190, China; ^3^Graduate University of Chinese Academy of Sciences, Beijing 100049, China

## Abstract

Protein identification is an integral part of proteomics research. The available tools to identify proteins in tandem mass spectrometry experiments are not optimized to face current challenges in terms of identification scale and speed owing to the exponential growth of the protein database and the accelerated generation of mass spectrometry data, as well as the demand for nonspecific digestion and post-modifications in complex-sample identification. As a result, a rapid method is required to mitigate such complexity and computation challenges. This paper thus aims to present an open method to prevent enzyme and modification specificity on a large database. This paper designed and developed a distributed program to facilitate application to computer resources. With this optimization, nearly linear speedup and real-time support are achieved on a large database with nonspecific digestion, thus enabling testing with two classical large protein databases in a 20-blade cluster. This work aids in the discovery of more significant biological results, such as modification sites, and enables the identification of more complex samples, such as metaproteomics samples.

## 1. Introduction

Proteomics is an emerging discipline based on the human genome project. Proteomics primarily aims to determine the presence and quantity of proteins. Similar to gene sequencing, the identification of protein sequences is important to facilitate a systematic understanding of key biological knowledge including protein structure, function, and evolutionary relationships. 

The basic principle of protein identification [1–3] based on mass spectrometry is to deduce protein amino acid sequences based on the mass/charge ratio of digested peptides and peptide fragments. The main strategies employed for protein identification by mass spectrometry include database searching, de novo sequencing, and peptide sequence tag. Among these strategies, database searching is the most popular. In this approach, experimental spectra are compared with theoretical spectra from database peptides to identify the best fit.

Commercial search engines such as Mascot [4] and SEQUEST [5] as well as open-source search engines such as X!Tandem [6], OMSSA [7, 8] and pFind (one of the first available protein identification software designed and developed in China) [9, 10] are very popular. With the rapid development of high-throughput biological mass spectrometry along with the exponential expansion of the protein database [11], computing technologies are presented with both an opportunity and a challenge to serve a notable function in solving biological problems. Commonly used protein identification software, such as Mascot, SEQUEST, and X!Tandem, currently possesses implemented cluster or cloud versions [12–17].

The main idea of parallel versions is to split the input spectra and to process each subset independently, which limits the identification speed to a stand-alone computer. Thus, problems arise once the index space becomes larger than the main memory size. Moreover, the types of modifications and the occurrence of digestion are unknown in most cases. If all modifications in the Unimod [18] database are specified, all search engines would not work properly. To complete the task within an acceptable duration under specific machine resources, including CPU frequency and memory size, protein identification has to employ restricted search, in which a small sequence database is used to specify a limited number of specific or semispecific enzymatic digestions as well as a limited number of commonly used modifications to limit the use of time and space. This condition makes most of the spectra produced by a mass spectrometer in proteomics experiments difficult to interpret. Only 5% to 30% of spectra can usually be identified [19]. An important reason is that several digestion methods and unknown or unexpected modifications likely exist. Thus, wrong candidate peptides will affect the subsequent protein identification process. Otherwise, the interpretation rate may double.

Thus, the development and analysis of a rapid identification method that can support a large protein database with any type of digestion and modification is urgently needed to facilitate protein deep analysis, like the expansion of metaproteomics [20].

Therefore, this paper designs and develops a distributed protein identification tool based on pFind to support nonspecific enzymatic digestion on a large database with unrestricted modification. The goal is to design and implement a practical, scalable, and efficient system to identify proteins rapidly and to identify more modification sites on a large protein sequence database. Actually, pFind has been proven more accurate than similar tools. In this work, we will focus on the acceleration of pFind to achieve greater identification speed. 

The remainder of the paper is organized as follows.  Section 2 describes the materials and methods used in this work.  Section 3 shows the evaluation of the proposed method and discusses the results. Finally, the conclusion and future work are given in Section 4.

## 2. Materials and Methods

In this section, we provide the background of open protein identification and discuss our optimization work.

### 2.1. Basic Principle and Trends

**Figure 1:**
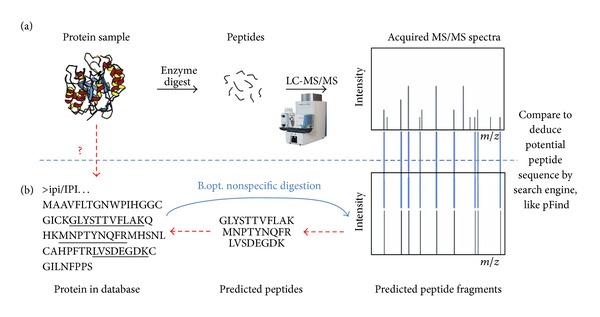
Principle of protein identification using MS/MS data.

Database searching is frequently employed to identify unknown amino acid sequences of peptides/proteins with high throughput. The main idea of this approach is shown in Figure 1. In this approach, proteins in the sample are digested into a peptide mixture. A mass spectrometer is then used to produce tandem mass (briefly as MS/MS) spectra, which are to be identified to query in a known protein database. On the other hand, the theoretical MS/MS spectra are predicted according to enzymatic digestion rules, that is, simulated digestion, based on peptide sequences from a known protein database. The most common method is to use a search algorithm to identify peptides by correlating experimental and theoretical MS/MS data generated from possible peptides in the protein sequence database through simulated digestion.

Simulated digestion theoretically refers to digestion based on the known protein sequences and enzyme specificity. Simulated digestion generally includes three types: specific, semispecific, and nonspecific. In specific digestion, protein sequence hydrolysis only occurs at a specific amino acid. For example, trypsin will cut polypeptide chain after lysine (K) or arginine (R) under the premise that proline (P) is not the next residue. In semispecific digestion, hydrolysis only occurs at some particular amino acids in one terminus, whereas the other will be disconnected at any amino acid. Nonspecific digestion occurs when disconnection occurs at any amino acid in both termini, that is, equivalent to any substrings of the amino acid sequence. Nonspecific digestion is usually avoided, especially when identifying on large databases, because of its running time and high memory demand.

Protein posttranslational modification on eukaryotic cells is of great significance in presenting the protein structure and function and explaining the mechanisms of major diseases. Over 1000 kinds of modifications are currently available in the database. Searching for an excessive number of modification types is thus unrealistic. Therefore, not more than 10 types of variable modification could be assigned for current mature search engines, such as SEQUEST and Mascot, which obviously cannot meet actual needs.

The types of digestion and modification are generally restricted. As the mainstream approach to database searching, the most significant advantage of the restricted method is its reduction of the scale of candidate peptides because this method assigns some factors depending on experience. However, individual experience is not always accurate. Despite appearing to be a perfect solution for the open method to support a large database, any type of digestion, and any type of modification, the search speed has restricted the development of this approach because of the large search space.

**Figure 2:**
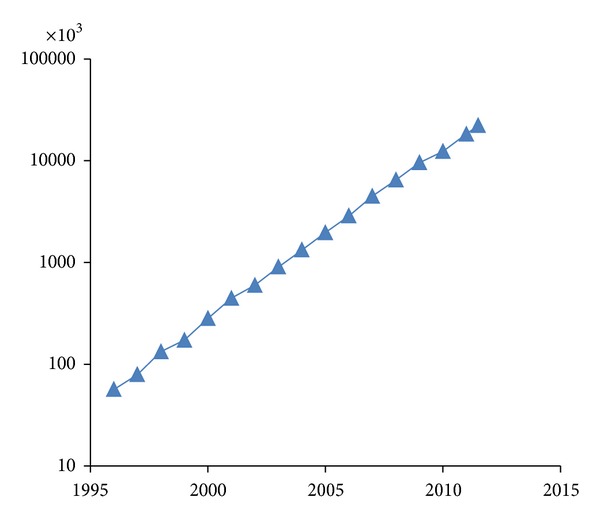
Increasing trend of protein database UniProtKB/TrEMBL (in entries).

Meanwhile, the exponential growth of the protein database, the rapid generation of mass spectrometry data, and the requirement for nonspecific digestion and postmodifications in complex-sample identification also pose a significant challenge on the identification scale and speed. The size of the genomic and protein sequence database grows exponentially, exceeding even Moore's Law in terms of the requirements for computing hardware. As shown in Figure 2, the increasing trend of the protein database UniProtKB/TrEMBL is a representative case.

The opinion of Patterson in [21] is curt and to the point: “…our ability to generate data now outstrips our ability to analyze it.”

### 2.2. Optimization Approaches

**Figure 3:**
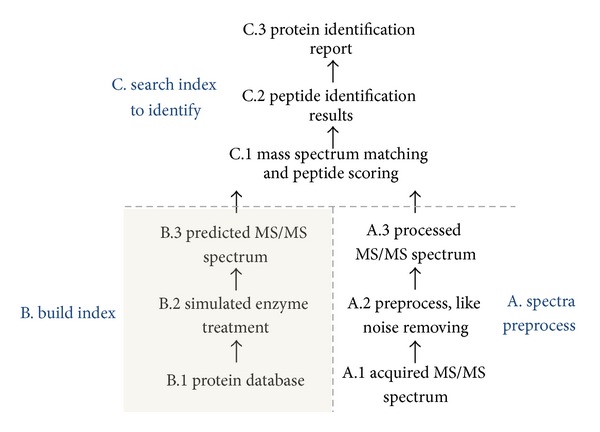
The typical workflow of protein identification by database searching.

Figure 3 shows the typical identification workflow, usually including (A.) spectra preprocess, (B.) build index, and (C.) search index to identify. 

Based on an analysis of the protein identification process, three main methods are available to accelerate search engines at present. First, preprocess protein database can be secured, such that a more efficient index structure can be constructed. This design is a high-performance solution in a small-scale protein database. However, for protein databases with a scale of tens of MB, the index created by this method has to use several GB of storage space. Moreover, building an index for a large database is time consuming. Second, efficient search algorithms or technologies can be presented for search engine acceleration, such as an inverted index. Third, a parallel search can be conducted to improve query efficiency in clusters.

With the popularity of cluster applications, successive parallel versions of some mainstream protein identification tools have been introduced. Most of these versions are based on the simple task of partitioning technology among spectra. As opposed to a stand-alone version, the identification speed can be increased several times. However, online digestion and fragmentation cannot be avoided for each retrieval.

To prepare for large-scale protein identification, identification on a large-scale protein database, with any type of restriction and modification, must be supported. Based on pFind, we designed a scalable and efficient system to meet the rapid identification needs.

pFind is one of the first protein identification software designed and developed in China. In terms of accuracy and speed, pFind has reached the level of international mainstream commercial software, such as SEQUEST and Mascot. As early as 2008, pFind has participated in the international evaluation on protein identification organized by the Association of Biomolecular Resource Facilities and has demonstrated strong performance in terms of identification accuracy and false positive rate control capability. pFind is currently the only protein search engine devoted to first-line research that was developed in China and is used by hundreds of groups around the world, including Duke University and MIT.

Search engines usually need to digest protein sequences online as well as filter peptides according to the mass error, which may add unnecessary overhead. When spectrum data are large, the overhead for online digestion will unnecessarily increase because the process will have to be performed repeatedly for each batch. If the index space can be guaranteed, nonspecific digestion on the protein database would significantly improve efficiency.

In nonspecific digestion, the protein sequence may cleave at any amino acid to form peptide fragments, which indicates that the hydrolysis of peptide can be any substring of the protein sequence. For each protein sequence, all subsequences within a specified length and mass range are generated. This optimization is step B.opt., as shown in Figure 1. Step B.2, as shown in Figure 3, can be handled in one way by nonspecific digestion for all enzymes and even offline. This condition not only lays the foundation for acceleration but also reduces the dependence on expertise.

In this work, we built a reverted index of peptide fragments generated by nonspecific digestion in mass prior to spectrum queries. The index generation process helps eliminate the overhead of simulated digestion during a search while naturally supporting the retrieval of nonspecific digestion. All subsequences generated from the protein database are sorted by their masses in ascending order, and an index table is constructed in which the key is the mass of each peptide represented by three integers: protein ID, start position, and amino acid length. Therefore, all index terms are recorded with equal lengths. Given an explicit mass or mass range to be queried, the time complexity of finding the first valid position in the datasheet is *O*(1). Some range searches obtained from spectrum peaks with mass tolerance can then quickly retrieve the unique index. Undoubtedly, this approach will simplify identification process and will save both the index build and search time.

Modification identification is another time-consuming process, and unrestricted posttranslational modification identification remains inadequate. InsPecT describes an unrestrictive PTM search algorithm that searches for any possible type of modification at once in a “blind search” mode, which does not depend on any given modification list. Such ideas can be used to identify more types of modifications, but its operation speed will be affected to a certain extent. By contrast, the number of modification types in Unimod is almost complete; at least in the vast majority of mass spectrometry experiments, Unimod is sufficient. As of Mascot version 2.3, support for Error Tolerant, an earlier proposed open identification method, is provided to iterate search over conventional identification. Moreover, this approach only supports semidigestion or all modifications in the Unimod database.

Using pFind with the DeltAMT algorithm [22], the Beijing Proteome Research Center identified core fucosylation (CF) modification. Over 100 CF glycoproteins and CF modification sites were identified from plasma samples of human liver cancer, the greatest number among all reports. The scale of identification results indicates significant progress in finding potential biomarkers. The discovery of a large number of modifications is of great significance for follow-up research and would aid in the early discovery of cancer markers [23]. Therefore, we have reasons to believe that accelerating pFind is an efficient method to accelerate postmodification prediction. 

The two-stage method can be used to determine one terminus of the peptide as well as to obtain a smaller number of candidates. The other terminus of the peptide can then be determined, taking the mass difference as the modification to seek in the inverted index of modification that was initially built according to Unimod, based on the smaller number of candidates. The complexity in determining one modification based on mass is *O*(1). To focus on acceleration, we will concentrate on dealing with nonspecific digestion.

### 2.3. Data Access Characteristics

In this section, we monitor the data usage of our open protein identification method and verify the robustness of this approach by implementing a measurable identification system based on Hadoop software.

**Figure 4:**
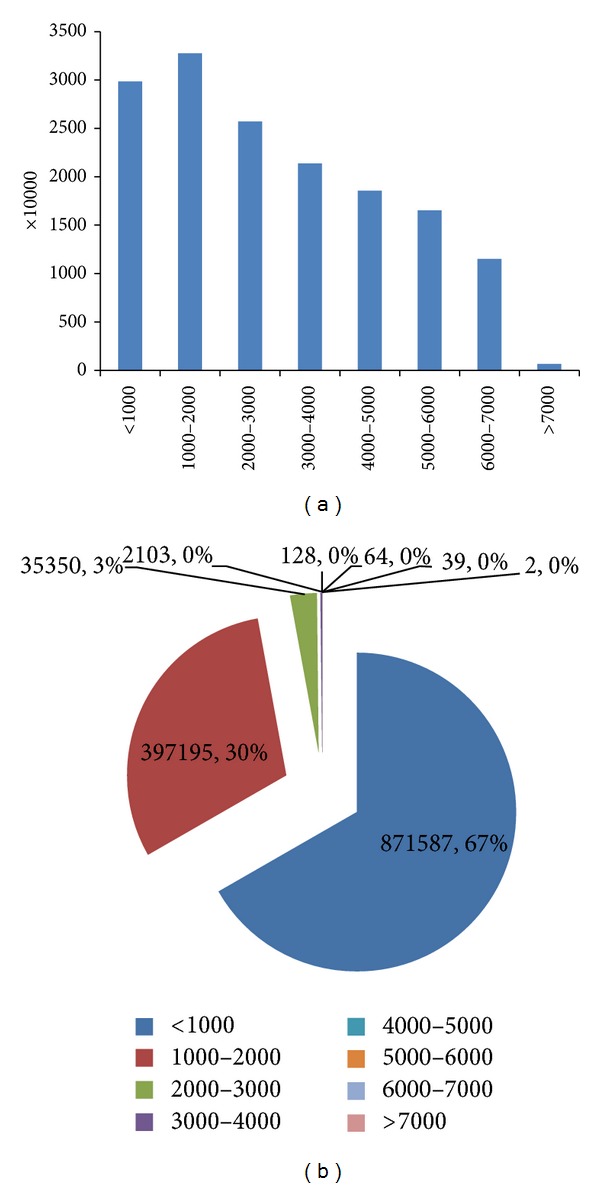
(a) Example of index distribution (*x*-axis is the mass range in Dalton, whereas *y*-axis is the statistic number). (b) Example of query distribution (legends are mass ranges in Dalton, and numbers shown in the pie include the statistic number and ratio).

We checked the index and query distributions and obtained the results shown in Figure 4. Basic analysis revealed that the query is mainly concentrated in less than 2000 Dalton; that is, 97% of total queries is within this range. However, only 40% of the data in the database is within 2000 Dalton. Meanwhile, the other 60% of the index will only account for only 3% of the queries, which is largely idle for these parts. The natural imbalance will inevitably cause some hot spots and low efficiency.

At first impression, the process of protein identification over a large-scale protein sequence database is similar to large-scale text information retrieval. Mature technologies in the large-scale Internet, such as Google GFS, map/reduce, and bigtable, can serve as references for protein identification. Such technologies also provide an important template for the construction of a large-scale protein identification cloud system with mechanisms such as distributed storage, load balance, and fault tolerance with the thousands-of-nodes cluster of Google.

However, the above results imply that success in the large-scale Internet with cloud system architecture is unsuitable for large-scale protein identification. This experiment is not similar in nature to experiments conducted using Amazon to distribute spectra [16, 17].

Nevertheless, we implement a cloud version of our approach with Apache Hadoop, a popular open-source software framework derived from Google techniques. This approach uses Hadoop map/reduce streaming to build the index for a protein database in advance. The database is then stored into the Hadoop HBase system for searching. The performance was just as expected.

### 2.4. Distributed Speedup

In this section, we design a distributed acceleration program for digestion-open protein identification based on the above analysis. To cope with the massive computational challenges brought by large-scale databases and nonspecific protein digestion, a parallel protein identification process must be efficiently implemented. As shown in Table 1, the number of peptide fragments generated by nonspecific digestion will increase by approximately 10,000 times compared with the protein number in conservative estimates. This number will potentially require a considerably large index space. Thus, a viable idea to self-design a system based on the map/reduce ideology to achieve unlimited expansion and to control data locality in the index arrangement.

To scale up support for identification on large protein databases, we introduce a method to partition and distribute the protein sequence database to build the index separately among cluster nodes that are parallel among proteins. With a large search space from nonspecific digestion, the index cannot be handled using the common stand-alone computer memory, like for ipi.HUMAN and UNIPROT-SPROT shown in Table 1, not saying a larger database and more complex analysis required. To address this issue, a simple and direct strategy is to partition the protein database into as many subsets as there are processors and then build the index in the memory of each subset independently. 

To scale up the system further, the database is partitioned according to the number of processors, and the subdatabase is again partitioned inside the processor according to the computer memory capacity. Within a node, the subdatabase can be partitioned efficiently. Partial identification is then conducted, and all results are collected as spectra. This process will help mitigate computer memory limitations. If speed is not an issue, the large database can be processed on any number of nodes. Moreover, the database is partitioned by accounting for the number of amino acids to balance the load. More detailed analysis can be referred to [24].

Latency hiding is another approach to develop scalable parallel machines. The main technology is data prefetching and multithreading. Data prefetching facilitates the prompt access or transfer of delayed data. Using multi-threading, computation and communication can be overlapped when a thread is computing to store data into the memory in preparation for the subsequent identification. Using this strategy, index-building time can be saved if the subblock index can be generated in advance to be prefetched when needed.

We distribute spectra among CPU cores in multithread to speed up the system. To improve the performance of the spectrum identification later, the spectra are sorted in mass before division into subtask blocks. Thereafter, mass spectra are assigned with close mass to the same sub-task. We achieved linear speedup in 320 processors with the spectrum distribution strategy on a small protein database [25]. Linear results were likewise obtained in 1024 processors in subsequent experiments.

In parallel computing, task scheduling is one of the most critical issues. The scheduling algorithm aims to achieve load balancing among compute nodes. In other words, parallel computing ensures that the tasks among compute nodes can be completed within close time points to minimize the overall running time. For simplicity, we only use the static scheduling according to the current application characterizations. In our experiments, static scheduling presents good performance in a homogeneous computer system.

## 3. Results and Discussion

In this section, we show some evaluation results in terms of identification speed and throughput of the distributed acceleration program on a 20-blade cluster. 

### 3.1. Linear Speedup

To evaluate the proposed approach, we tested a 20-blade cluster with an Intel two-quad-core CPU and 8 GB memory. We took the two databases mentioned in Table 1 and a raw file randomly sampled from TTE experiments as input. The raw file was obtained from LTQ-Orbitrap Velos with HCD collision. To save test time, we regularly sampled 1096 spectra as a test case.

**Figure 5:**
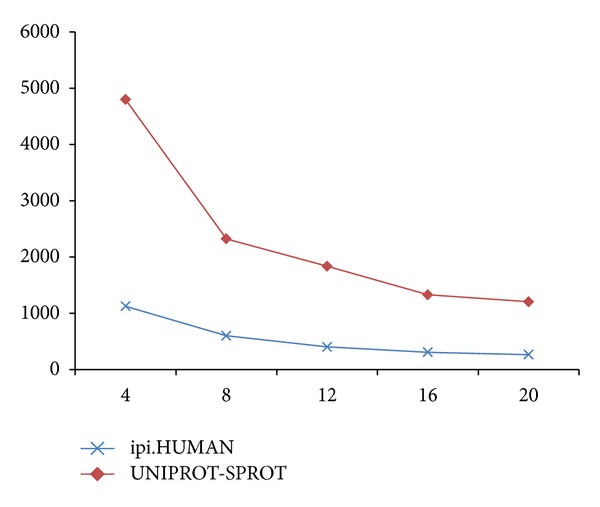
Performance test on the distributed identification system (no. of nodes—seconds).

Figure 5 shows good scalability in the near linear area from 4 to 20 nodes on both ipi.HUMAN and UNIPROT-SPROT. In our analysis, the system can easily be scaled up to support a larger database. Users can add new nodes in the cluster to meet the growing demand.

### 3.2. Support for Real-Time Identification over Large Protein Databases

**Figure 6:**
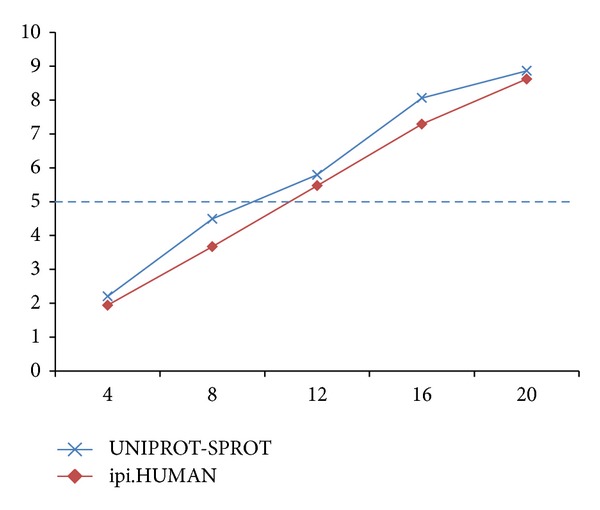
Identification throughput in spectra/s/100M B (no. of nodes—spectra/s/100 MB).

In analyzing the system overhead, we found that over 70% of time is used to build the index. We thus consider building the index offline with nonspecific digestion support and then perform partial prefetching when needed. Simulated digestion is only required once for each protein sequence database if the index is stored. When an index block is used for identification, the next block can be fetched by another process in background to prepare for the next round. Given that prefetching time can overlap, that is, the part B overhead in Figure 3 can be saved, nearly four times of improvement can be achieved. Considering LTQ as an example, the 16-node system is sufficient to support real-time identification on it, which generates approximately 5 MS/MS spectra per second. These results in Figure 6 show that real-time identification is feasible.

## 4. Conclusion

Motivated by the significance of protein identification, we identify the considerable computation demand primarily based on the development of high-throughput spectrometry and the expansion of known protein databases. We then propose an open identification method to support nonspecific digestion, which lays the foundation for acceleration and reduces the dependence on digestion expertise. We likewise accelerated the identification speed to real-time through appropriate distribution.

In the future, we will still focus on improving the speed and throughput of protein identification using algorithms and workflows. Moreover, we plan to conduct more work on open identification. We will continue contributing to practical protein identification systems similar to Google for large-scale Internet.

## Figures and Tables

**Table 1:** Scale change in nonspecific digestion.

DB	Version	Fasta size (MB)	No. of proteins	No. of peptide fragments	Index size (GB)
ipi.HUMAN	3.87	48.71	91,491	1,939,985,477	44.44
UNIPROT-SPROT	201108	240.51	532,234	9,964,797,926	235.51

Note: peptide mass is 300–8000 Dalton. Peptide length is 3–60 amino acids.

## References

[1] Cottrell JS (2011). Protein identification using MS/MS data. *Journal of Proteomics*.

[2] Johnson RS, Davis MT, Taylor JA, Patterson SD (2005). Informatics for protein identification by mass spectrometry. *Methods*.

[3] Edwards NJ (2011). Protein identification from tandem mass spectra by database searching. *Methods in Molecular Biology*.

[4] Perkins DN, Pappin DJC, Creasy DM, Cottrell JS (1999). Probability-based protein identification by searching sequence databases using mass spectrometry data. *Electrophoresis*.

[5] Eng JK, McCormack AL, Yates JR (1994). An approach to correlate tandem mass spectral data of peptides with amino acid sequences in a protein database. *Journal of the American Society for Mass Spectrometry*.

[6] Craig R, Beavis RC (2004). TANDEM: matching proteins with tandem mass spectra. *Bioinformatics*.

[7] Geer LY, Markey SP, Kowalak JA (2004). Open mass spectrometry search algorithm. *Journal of Proteome Research*.

[8] Geer LY Reducing false positive rates in MS/MS sequence searching and incorporating intensity into match based statistics. ftp://ftp.ncbi.nih.gov/pub/lewisg/presentations/asms06poster.pdf.

[9] Li D, Fu Y, Sun R (2005). pFind: a novel database-searching software system for automated peptide and protein identification via tandem mass spectrometry. *Bioinformatics*.

[10] Wang L, Li D, Fu Y (2007). pFind 2.0: a software package for peptide and protein identification via tandem mass spectrometry. *Rapid Communications in Mass Spectrometry*.

[11] EBI http://www.ebi.ac.uk/uniprot/TrEMBLstats/.

[12] Bjornson RD, Carriero NJ, Colangelo C (2008). X!!Tandem, an improved method for running X!Tandem in parallel on collections of commodity computers. *Journal of Proteome Research*.

[13] Duncan DT, Craig R, Link AJ (2005). Parallel tandem: a program for parallel processing of tandem mass spectra using PVM or MPI and X!Tandem. *Journal of Proteome Research*.

[14] Quandt A, Hernandez P, Kunzst P (2007). Grid-based analysis of tandem mass spectrometry data in clinical proteomics. *Studies in health technology and informatics*.

[15] Zosso D, Podvinec M, Müller M, Aebersold R, Peitsch MC, Schwede T (2007). Tandem mass spectrometry protein identification on a PC grid. *Studies in Health Technology and Informatics*.

[16] Halligan BD, Geiger JF, Vallejos AK, Greene AS, Twigger SN (2009). Low cost, scalable proteomics data analysis using Amazon’s cloud computing services and open source search algorithms. *Journal of Proteome Research*.

[17] Pratt B, Howbert JJ, Tasman NI, Nilsson EJ (2012). Mr-tandem: parallel x!Tandem using hadoop MapReduce on Amazon web services. *Bioinformatics*.

[18] Unimod http://www.unimod.org/.

[19] Ahrné E, Müller M, Lisacek F (2010). Unrestricted identification of modified proteins using MS/MS. *Proteomics*.

[20] Wilmes P, Bond PL (2006). Metaproteomics: Studying functional gene expression in microbial ecosystems. *Trends in Microbiology*.

[21] Patterson SD (2003). Data analysis—the Achilles heel of proteomics. *Nature Biotechnology*.

[22] Fu Y, Xiu L, Jia W (2011). DeltAMT: a statistical algorithm for fast detection of protein modifications from LC-MS/MS data. *Molecular and Cellular Proteomics*.

[23] Jia W, Lu Z, Fu Y (2009). A strategy for precise and large scale identification of core fucosylated glycoproteins. *Molecular and Cellular Proteomics*.

[24] Zhang W, Chi H, Lu Y, Huang Y, Zhao X, He S Preliminary search engine for open protein identification.

[25] Wang L, He S, Sun R (2010). An efficient parallelization of phosphorylated peptide and protein identification. *Rapid Communications in Mass Spectrometry*.

